# Elderly Onset of Weakness in Facioscapulohumeral Muscular Dystrophy

**DOI:** 10.1155/2012/726984

**Published:** 2012-09-18

**Authors:** Dominic B. Fee

**Affiliations:** Department of Neurology, University of Kentucky Chandler Medical Center, Lexington, KY 40536, USA

## Abstract

A 77-year-old male is presented. He had onset of proximal weakness 10 years earlier. His course was slowly progressive. Despite having phenotypic features of facioscapulohumeral muscular dystrophy (FSH), genetic testing for this was delayed because of his age of onset, lack of family history, and benign appearing muscle biopsy. This case is one of the oldest onset of weakness in genetically confirmed FSH and highlights the recognized expansion in phenotype that has occurred since the advent of genetic testing.

## 1. Introduction

Facioscapulohumeral Muscular Dystrophy (FSH) is the third most common muscular dystrophy. It predominately results in weakness of facial, shoulder, and upper arm muscles with onset of symptoms in most individuals prior to age 20-year-old (yo). Around 95% of individuals with FSH have a deletion in the 3.3 kb D4Z4 repeat segment on chromosome 4; interestingly, and confusing for assessing this, the deletion is only pathologic with hypomethylation of DNA, occurring on specific D4Z4 alleles and having at least one repeat present [1, 2]. With the advent of genetic testing, the accepted phenotypic expression has expanded [2, 3]. However, there are still patients who challenge the notion of what is an accepted initial presentation for FSH.

This paper describes a patient who had onset of weakness in his seventh decade of life and had no dystrophic changes on muscle biopsy. Because of his pattern of weakness, FSH genetic testing was performed and confirmed the diagnosis. This is one of the oldest age of symptom onset reported in a genetically confirmed FSH patient [4–6].

## 2. Case Report

 The patient presented at 77-year-old with an approximately ten-year history of slowly progressive shoulder and proximal arm weakness and one-year history of speaking and swallowing difficulties with rare dyspnea on exertion. Throughout his life he was physically very active including military service (National Guard) until retiring at age 60-year-old, outdoor activities, karate, and violin. At 65-year-old, he stopped karate because of concern of injury from sparring with much younger individuals; at that same age he also stopped playing violin because of poor finger control. It was not, though, until a few years later that he developed his presenting complaints.

 His past medical history was significant for mild-type II diabetes mellitus, recent gastrointestinal bleed, coronary artery disease with stenting three years prior to presentation, cataracts, and bilateral knee replacements four years prior to presentation. Medications included simvastatin, aspirin, metoprolol, niacin, lansopraxole, metformin, metoclopramide, as needed nitroglycerin, and various vitamins and minerals. His family history was significant for coronary artery disease on the paternal side and diabetes mellitus on the maternal. There is no family history of early onset muscle weakness. Other aspects of his social history and review of systems were unremarkable.

 On physical exam, his vitals and general examination were unremarkable. On neurological examination, mental status, reflex, and sensory examinations were appropriate for his age. On cranial nerve examination, the patient had dysarthric speech, predominately slurred, and bilateral facial weakness, 4/5 on the Medical Research Council (MRC) scale unable to fully purse lips. There was no worsening of weakness with sustained activity. Other aspects of the cranial nerve examination were unremarkable. Motor examination revealed weakness predominately proximal and in the upper extremities. Shoulder abduction was 4−/5, rest of the upper extremity was 4+/5, hip flexion 4−/5, and rest of lower extremities was 5−/5 on the Medical Research Council (MRC) scale. There was atrophy of the shoulder girdle, mild atrophy of intrinsic muscles of his feet, and decreased tone throughout. His gait was broad based and slow.

 Testing done one year prior to presentation was significant for myopathic, short duration with low amplitude, units in proximal, upper extremity muscles on nerve conduction studies/electromyography testing (NCS/EMG), borderline creatine kinase (250 U/L, normal (n) 150–230 U/L), serial swallowing studies demonstrating new onset difficulties, and degenerative changes appropriate for age on magnetic resonance imaging (MRI) of the cervical spine. The following were unremarkable-acetylcholine receptor antibodies, thyroid function tests, complete metabolic panel, erythrocyte sedimentation rate, serum protein electrophoresis, and MRI of the brain. Chest X-ray was significant for flattened hemidiaphragms, scattered calcifications, and linear scarring; the changes were concerning for chronic obstructive pulmonary disease and were unchanged over four years. Cardiac consultation including echocardiogram and catherization was unremarkable. The referring neurologist was already concerned for a muscular dystrophy.

**Figure 1:**
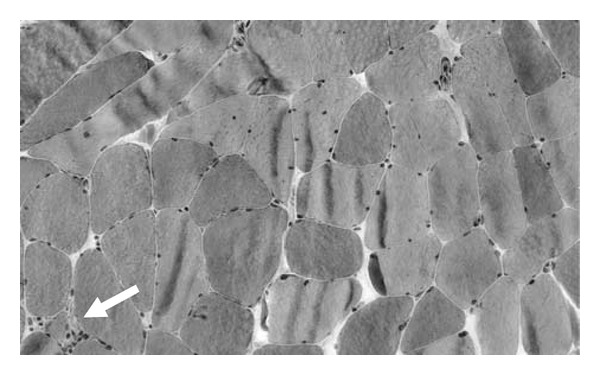
Trichrome stained muscle biopsy, 20x magnification lens. The sole degenerating fiber in the entire biopsy is in the bottom left corner (arrow). There are no dystrophic changes present.

 At presentation, pulmonary function testing (PFTs) and a muscle biopsy were ordered. The PFTs were unremarkable (FVC 98% predicted, FEV1 91% predicted). The muscle biopsy of the deltoid was similarly unremarkable; there was a single degenerating myofiber with lymphocytic infiltration, a couple areas of minor, perivascular inflammation, and a few muscle fibers with increased internal nuclei, Figure 1. His simvastatin was halted without any improvement. A trial of prednisone was initiated and halted when it leads to worsening symptoms. Four months after presentation, repeat testing was performed. An NCS/EMG study including repetitive stimulation was similar to prior studies; there were some changes on the EMG aspect suggesting proximal myopathy. The following blood work was unremarkable antinuclear antibodies, erythrocyte sedimentation rate, complete blood count, creatine kinase, rheumatoid factor, lactate, immunofixation, parathyroid hormone panel, pyruvate, hemoglobin A1c, thyroid stimulating hormone; a complete metabolic panel had elevated glucose (still tapering prednisone) and genetic testing for FSH which was abnormal (one allele with 30 kb, other > 48 kb; nl > 42 kb, pathologic 12–37 kb). Based on the genetic testing a diagnosis of FSH was made. Although his pattern of weakness at presentation suggested FSH, his age of onset suggested another etiology being present. The lack of dystrophy changes on the muscle biopsy with some nonspecific inflammatory changes prompted the steroid trial for possible polymyositis.

 The patient had progressive speech, swallowing, and breathing problems. He had an unremarkable esophagogastroduodenoscopy. Pulmonary consultation including sleep study felt that a central hyperventilation syndrome was present, given an arterial blood gas with low PCO2 (26.6 mm HG, nl 35–45), with nl PH and PO2 and stable pulmonary function testing—FVC 97% predicted and FEV1 95% predicted. A trial of paroxetine for the hyperventilation syndrome was unrewarding. The combination of pyridostigmine and oral albuterol did lessen the shortness of breath; physical therapy improved extremity functioning.

 The patient continued with a slow decline until three years after presentation when he had a myocardial infarction requiring stenting. His cardiac ejection fraction declined to 25%; it was 51% three years prior; it subsequently improved to 40%. His breathing dramatically worsened, but his oxygen saturation on room air remained above 92%. Eight months after this event, he had another cardiac event with a precipitous decline. He became wheelchair bound, required percutaneous endoscopic gastrostomy tube to prevent aspiration, and became supplemental oxygen dependent. A little over four years after presentation, the patient died from cardiopulmonary complications.

## 3. Discussion

 Typically, 95% of patients with FSH have signs and symptoms by 20 years of age and have dystrophic changes on muscle biopsy [3]. This patient did not. Obviously with the advent of genetic testing, individuals have been confirmed to have FSH who have features desperate from the typical phenotype.

 One of the current thrusts of investigating genetic conditions is why there are significantly different phenotypes. Two simple explanations are different environmental exposures and different mutation in the same gene resulting in different protein shape/function and expression. However, it is also felt that there are additional genes that can modify expression. Investigating these in FSH has been very difficult, mainly, because the specific genetic abnormality has only recently been identified [1].

 The patient's age and benign appearing biopsy suggest that modifying genes are present which affect expression beyond the repeat length differences. It is unknown what precisely causes dystrophic changes in muscular dystrophies and what is the correlation between dystrophic changes and phenotype; however, this patient lacked any evidence of dystrophic changes despite being in his eighth, nearing ninth, decade of life. His benign appearing muscle biopsy did delay diagnosis which put him at risk from complications of a therapeutic trial of prednisone.
